# Association Between NAT2 Polymorphism and Lung Cancer Risk: A Systematic Review and Meta-Analysis

**DOI:** 10.3389/fonc.2021.567762

**Published:** 2021-03-11

**Authors:** Ke Zhu, Aiqun Xu, Wanli Xia, Pulin Li, Binbin Zhang, Huihui Jiang, Sijing Zhou, Ran Wang

**Affiliations:** ^1^ Department of Respiratory and Critical Care Medicine, The First Affiliated Hospital of Anhui Medical University, Hefei, China; ^2^ Department of General Medicine, Hefei Second People’s Hospital, Hefei, China; ^3^ Department of Thoracic Surgery, The First Affiliated Hospital of Anhui Medical University, Hefei, China; ^4^ Department of Occupational Medicine, Hefei Third Clinical College of Anhui Medical University, Hefei, China; ^5^ Department of Occupational Medicine, Hefei Prevention and Treatment Center for Occupational Diseases, Hefei, China

**Keywords:** NAT2, genetic polymorphism, lung cancer, systematic review, phenotype

## Abstract

Lung cancer is the leading cause of cancer-related death worldwide and has a high incidence rate. N-Acetyltransferase 2 (NAT2) is a polymorphic xenobiotic enzyme, which can catalyze N-acetylation and O-acetylation of various carcinogens such as aromatic, heterocyclic amines and hydrazines. At present, many studies have explored the effects of NAT2 polymorphism on lung cancer, but we found inconsistent results. We researched 18 published studies, involving 4,016 patients and 5,469 controls, to more accurately assess the effects of NAT2 polymorphism on lung cancer risk and to investigate whether smoking is associated. We used STATA software to analyze the extracted data and used STATA for subgroup analysis, sensitivity analysis, and to perform publication bias tests. To determine the correlation, we used the crude odds ratio (ORs) with 95% confidence interval (CIs). Our study was prospectively registered in PROSPERO (CRD42020159737). The odds ratio was 1.53 (95% CI: 1.21**–**1.95, I² = 45.2%, P=0.104) for the NAT2 slow + intermediate phenotype ***versus*** rapid phenotype. The results suggested that people with NAT2 non-rapid (slow + intermediate) phenotype have a significantly increased risk of lung cancer. In addition, NAT2 rapid phenotype was significantly associated with reduced risk of lung cancer, compared with slow phenotype or intermediate phenotype (slow phenotype ***vs***. rapid phenotype: OR: 1.61, 95% CI: 1.07–2.42, I²= 50%, P= 0.075; intermediate phenotype ***vs***. rapid phenotype: OR: 1.47, 95% CI: 1.15–1.88, I²= 40.3%, P= 0.137).

## Introduction

Lung cancer is the leading cause of cancer-related death worldwide and has a high incidence rate (1, 2). Lung cancer is mainly caused by smoking and other environmental factors, such as exposure to heavy metals, radiation, asbestos, and air pollution (3). However, even if exposed to these factors, only a small portion of people suffer from lung cancer, which indicates that genetic factors also play an important role in the occurrence of lung cancer (4). The current view is that the occurrence and development of lung cancer is the result of the interaction between environment and heredity (4, 5).

N-Acetyltransferase 2 (NAT2) is a polymorphic xenobiotic enzyme, which can catalyze N-acetylation and O-acetylation of various carcinogens such as aromatic, heterocyclic amines, and hydrazines (6). More than 27 variants or combinations of single nucleotide polymorphisms (SNPs) have been found in NAT2 (7). Different SNPs combinations can produce different alleles, resulting in NAT2 slow, intermediate, and rapid acetylation phenotype (8). The alleles of NAT2 and the corresponding phenotype can be found on the authoritative NAT2 organization website “http://nat.mbg.duth.gr/Human%20NAT2%20alleles_2013.htm#_Footnotes.” NAT2, individuals homozygous for rapid alleles, such as NAT2*4, NAT2*11A, NAT2*12A, NAT2*12B, NAT2*12C, NAT2*13, are identified as rapid genotype (9). Individuals homozygous for slow alleles, such as NAT2*5, NAT2*6, NAT2*7, are identified as slow genotype (9). Individuals with one rapid allele and one slow allele are classified as intermediate acetylator phenotype (9, 10).

At present, many studies have explored the association between NAT2 polymorphism and lung cancer, but we found inconsistent results (11–28). We conducted a meta-analysis of 18 published studies, involving 4,016 patients and 5,469 controls, to more accurately assess the Effects of NAT2 polymorphism on lung cancer risk.

## Materials and Methods

In this meta-analysis, we followed the Preferred Reporting Items of the Systematic Reviews and Meta-analysis (PRISMA) guidelines and the Cochrane Handbook of Systematic Reviews of Interventions. The protocol for our study could be found in the PROSPERO under registration id CRD42020159737. The research issues in the systematic review were clearly defined by population, interventions, comparators, outcomes, and study design (PICOS). The population is the patients with lung cancer, intervention and comparator are phenotypes of NAT2 polymorphism, the outcome is odds ratios (ORs), and the study design is a case-control study.

### Search Strategy

We used the following electronic bibliographic databases: PubMed, EMBASE, and Web of Science. There were no limits on the search. Search terms were roughly divided into six categories: N-acetyltransferase 2, NAT2, genetic polymorphism, pulmonary neoplasm, lung neoplasm, and lung cancer. We used EndNote (X9, https://endnote.com/) to weed out duplicate articles, and then we read the titles and abstracts of all articles we retrieved to exclude meta-analyses, review articles, case reports, meeting minutes, and irrelevant articles.

The inclusion criteria included :

1. Case-control or cohort or nest case-control studies focused on the role of NAT2 acetylation status in lung cancer risk.
2. Studies on the acetylation state of NAT2, including the rapid acetylation phenotype, intermediate acetylation phenotype, and slow acetylation phenotype.
3. The required information was extracted from the case group and control group (different NAT2 acetylation states).
4. Those with sufficient data to calculate the odds ratio (OR).

The exclusion criteria included:

1. No control group
2. Lack of statistics on the acetylation status of NAT2
3. Case reports, meta-analyses, review articles, meeting minutes, and irrelevant articles
4. Experiments on animals
5. Unable to find the full text

### Data Extraction

We selected two independent investigators who were co-authors of this meta-analysis to screen the search results. After they found relevant articles, they searched the full text of those studies, read them carefully, and evaluated them to decide whether to include or exclude them. The whole process was done by two investigators independently. If there was any disagreement over whether to include an article, they would discuss it with a third reviewer to decide whether to include the article. The first author, year of publication, country of origin, the ethnicity, sources of controls, smoking status (i.e., smoking *vs*. non-smoking status) in the case and the control groups, genotyping method, histological type, the sample size of cases and controls, numbers of cases, and controls with different NAT2 phenotypes were the data types extracted from each study. The Newcastle-Ottawa Scale (NOS) was the method we evaluate the quality of the included studies (29). Each study was graded on this basis (30). The quality evaluation of the included studies was also carried out independently by two researchers. The NOS evaluation was carried out from three categories: selection of study sample, comparability between case and control group, and outcome assessment (31). The total score for each study is 0-9 (31). If there was any disagreement, they would discuss with a third reviewer to properly evaluate the included articles. To minimize the bias, we included studies with a NOS score of 5 and above.

### Statistical Analysis

In almost all of the included studies, the effect of NAT2 polymorphism on lung cancer risk was estimated using crude odds ratios (ORs) of 95% confidence intervals (CIs). We first evaluated the pooled ORs of the primary model (slow + intermediate phenotype *vs*. rapid phenotype) (8). Then, we evaluated the pooled ORs of the recessive model (slow phenotype *vs*. intermediate + rapid phenotype) (8). In addition, the pooled ORs of slow *vs*. intermediate phenotype, intermediate *vs*. rapid phenotype, and slow *vs*. rapid phenotype were also estimated (8). We used χ²-based Q statistical to test heterogeneity. I² and P values were used to quantify the impact of heterogeneity (32). If heterogeneity was not obvious (I²<50% and P-value is > 0.10), we used the fixed-effects model. Otherwise, we used the random-effects model (33). After that, we used the method of sensitivity analysis to assess the stability of the result. We used Begg’s funnel plots and Egger’s tests to evaluate publication bias and we used the STATA software (version 12.0, Stata Corporation, College Station, TX) to conduct the statistical analyses (34, 35).

## Results

### Study Screening Results

**Figure 1:**
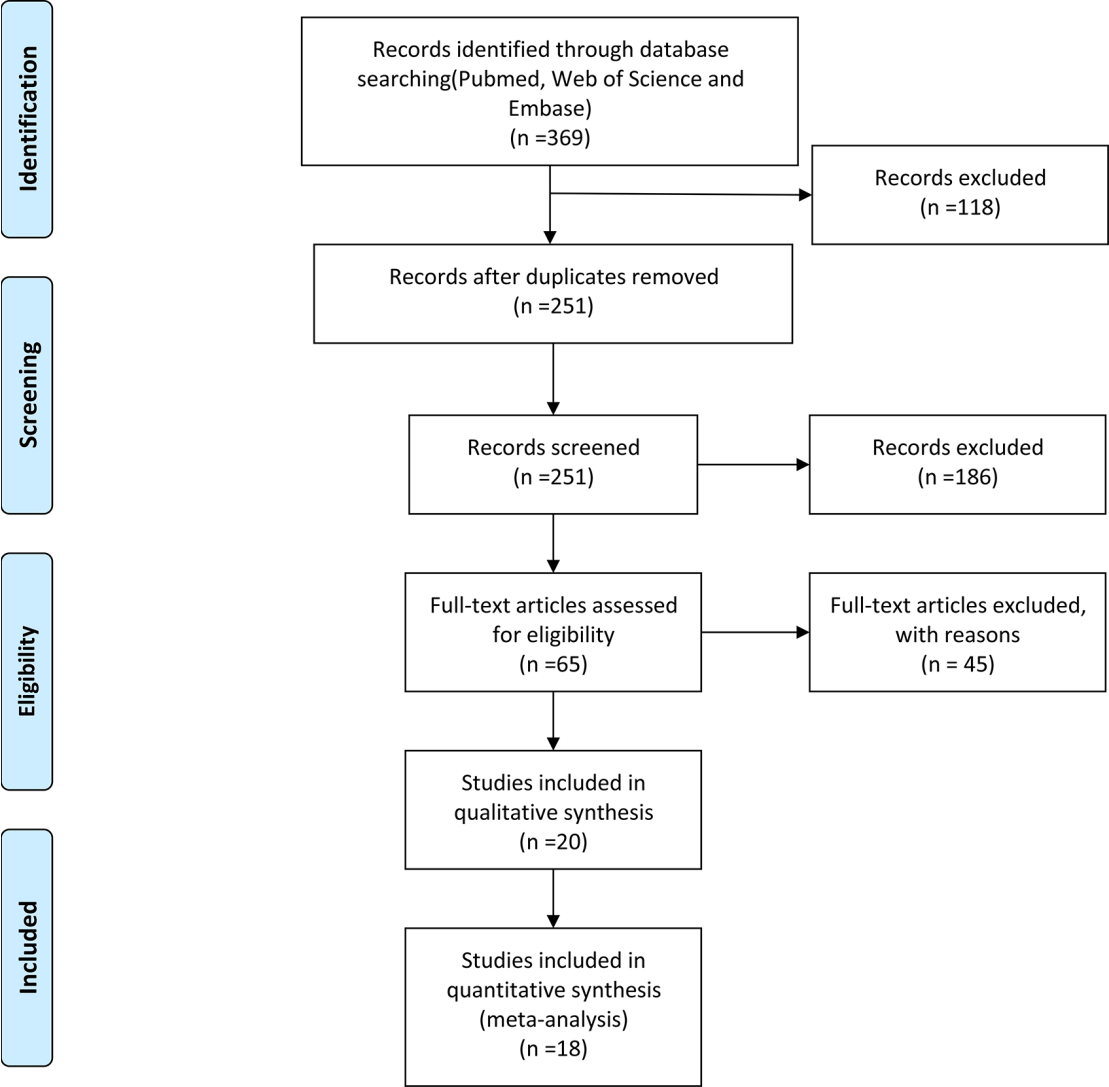
The study selection process of this meta-analysis.

In **Figure 1**, 369 studies were selected through PubMed, EMBASE, and Web of Science, with the help of the EndNote software, we excluded 118 repetitive articles. The specific search strategy is shown in **Table S1**. Reading the titles and abstracts of the articles, we found that 186 articles were irrelevant. The full text of four studies was not found. Besides, we excluded 41 articles due to irrelevant content. Among these, 34 articles did not assess the acetylation status of NAT2, and 7 articles were reviews and meta-analyses. The Newcastle-Ottawa Scale (NOS) is the method we used to assess the quality of the remaining included studies. **Table S2** shows the NOS scores for each study. Finally, 18 studies met our criteria, including 4,016 patients and 5,469 controls.

The major information we collected from these articles is shown in **Table 1**. The data collected included first author, year, country, ethnicity, source of controls, genotyping means, and phenotype distribution. The study by Sorensen M et al. was a nested case-control study, while the other studies were case-control studies. Besides, there were eight hospital-based studies, eight population-based studies, and one hospital and population-based study. In terms of patient backgrounds, 10 studies included European populations, and 8 included Asian populations. Of the genotyping methods, only one used TaqManSNP (TaqMan) (20).

**Table 1:** The data types extracted in this research.

First author	Year	Country	Ethnicity	Source of controls	Genotyping method	Slow^c^ (case)	Rapid^c^ (case)	Slow^c^ (control)	Rapid^c^ (control)
Mota^11^	2015	Portugal	European	HB^a^	PCR–RFLP	153	36	168	61
Zhang^12^	2014	China	Asian	PB^b^	PCR–RFLP	135	795	113	887
Mahasneh^13^	2012	Jordan	Asian	HB	PCR–RFLP	22	27	61	38
Zupa^14^	2009	Italy	European	HB	PCR–RFLP	43	32	50	71
Sobti^15^	2009	India	Asian	HB	PCR–RFLP	50	252	33	269
Lee^16^	2009	China	Asian	PB	PCR–RFLP	22	95	36	83
Osawa^17^	2007	Japan	Asian	HB	PCR–RFLP	14	99	9	112
Chen^18^	2006	China	Asian	PB	PCR–RFLP	18	79	43	154
Chiou^19^	2005	China	Asian	PB	PCR–RFLP	27	135	64	144
Sorensen^20^	2005	Denmark	European	unknown	TaqMan PCR-RFLP	156	99	143	121
Skuladottir^21^	2005	Denmark, Norway	European	PB	PCR–RFLP	154	87	321	219
Belogubova^22^	2005	Russia	European	HB, PB	PCR–RFLP	99	79	426	289
Wikman^23^	2001	German	European	HB	PCR–RFLP	237	151	196	149
Hou^24^	2000	Norway	European	PB	PCR–RFLP	169	112	237	138
Saarikoski^25^	2000	Finland	European	PB	PCR–RFLP	102	93	152	140
Seow^26^	1999	China	Asian	HB	PCR–RFLP	60	93	36	105
Nyberg^27^	1998	Sweden	European	PB	PCR–RFLP	113	70	96	62
Martinez^28^	1995	Spain	European	HB	PCR–RFLP	61	47	136	107

a Hospital-based study.

b Population-based study.

c Phenotype distribution: slow represents the slow acetylation status and rapid represents the rapid + intermediate acetylation status.

### Meta-Analysis

**Figure 2:**
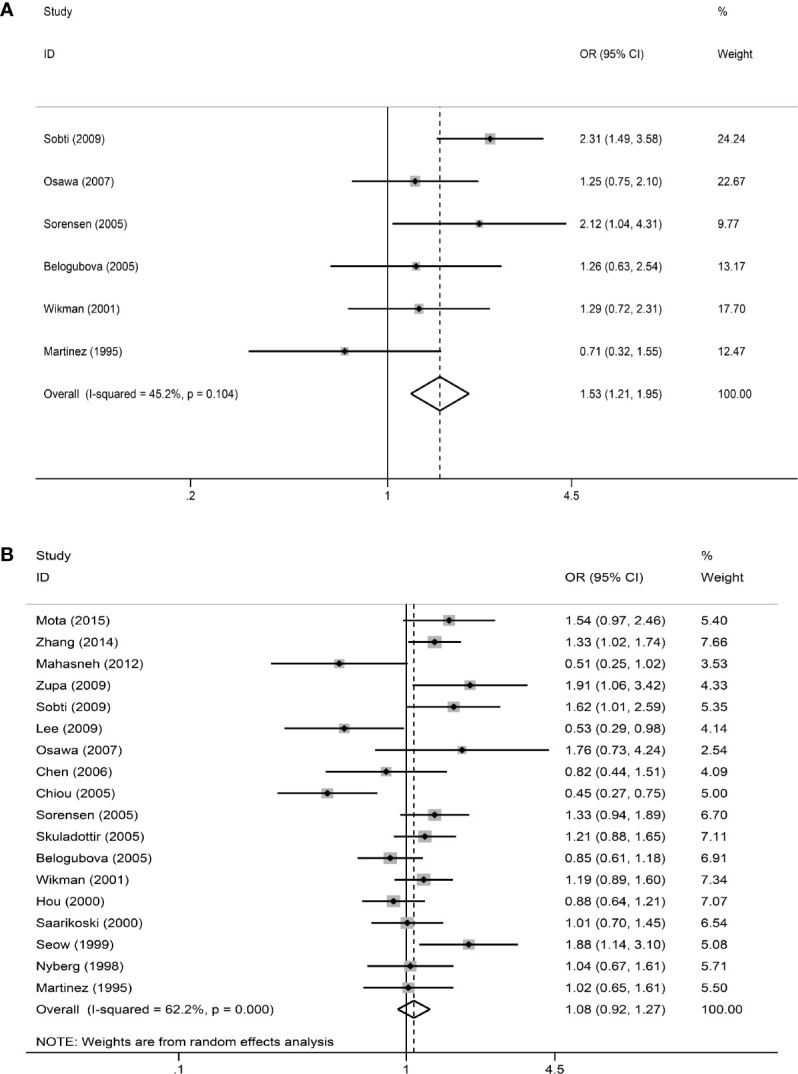
The meta-analysis results. **(A)** Meta-analysis for the association between NAT2 polymorphism and lung cancer risk (slow + intermediate *versus* rapid). OR is represented as a square and its 95% CI estimate is represented as two horizontal lines. The weight has reflected the area of the square (inverse variance). The diamond represents the combined results of all the studies. **(B)** Meta-analysis for the association between NAT2 polymorphism and lung cancer risk (slow *versus* intermediate + rapid).

**Figure 3:**
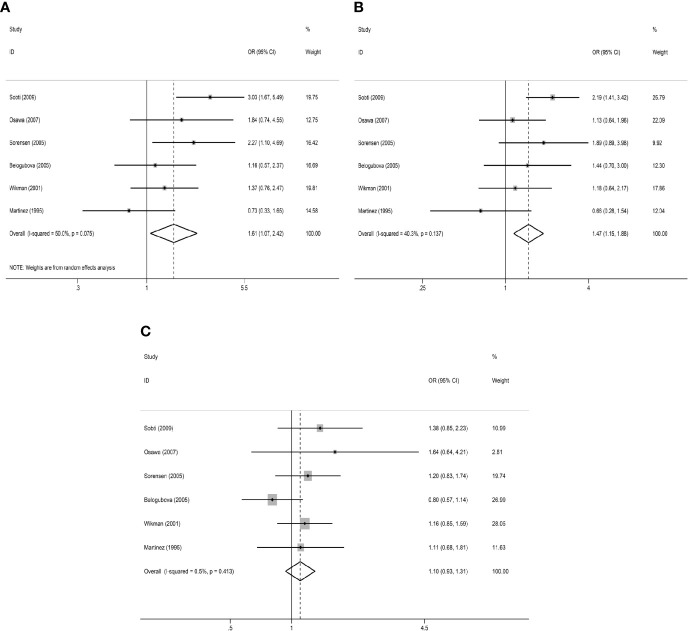
Results of comparison of three NAT2 acetylator statuses. **(A)** Meta-analysis for the association between NAT2 polymorphism and lung cancer risk (slow *versus* rapid). **(B)** Meta-analysis for the association between NAT2 polymorphism and lung cancer risk (intermediate *versus* rapid). **(C)** Meta-analysis for the association between NAT2 polymorphism and lung cancer risk (slow *versus* intermediate).

In the primary model (slow + intermediate phenotype *vs*. rapid phenotype), the combined OR value of the six studies (15, 17, 20, 22, 23, 28) was 1.53 (95% CI: 1.21–1.95, I²= 45.2%, P=0.104 for heterogeneity based on a fixed-effects analysis model). The results suggested that people with NAT2 non-rapid (slow + intermediate) phenotype have a significantly increased risk of lung cancer. The results are shown in **Figure 2A**. As shown in **Figure 2B**, all 18 studies could be included in the analysis of the recessive model, but there was no significant difference between slow phenotype and non-slow (intermediate + rapid) phenotype (OR: 1.08, 95% CI: 0.92–1.27, I²= 62.2%, P<0.001 for heterogeneity based on a random-effects analysis model). The six studies included in the primary model (15, 17, 20, 22, 23, 28) were also included to compare the three NAT2 acetylator statuses. As shown in **Figure 3**, NAT2 rapid phenotype was significantly associated with reduced risk of lung cancer, compared with slow phenotype or intermediate phenotype (slow phenotype *vs*. rapid phenotype: OR: 1.61, 95% CI: 1.07–2.42, I²= 50%, P= 0.075; intermediate phenotype *vs*. rapid phenotype: OR: 1.47, 95% CI: 1.15–1.88, I²= 40.3%, P= 0.137). However, there was no significant difference between slow phenotype and intermediate phenotype (OR: 1.10, 95% CI: 0.93–1.31, I²= 0.5%, P= 0.413).

**Figure 4:**
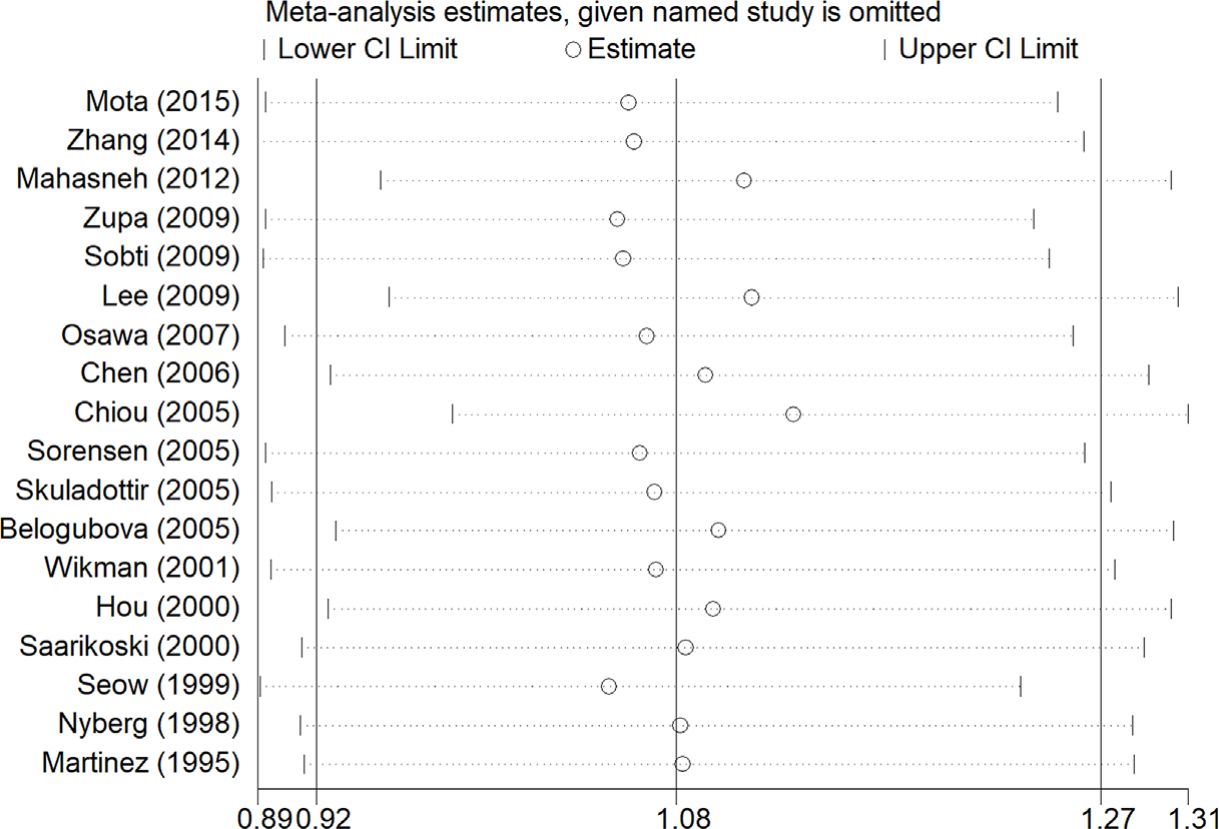
Results of the sensitivity analysis. The middle vertical line represents the combined OR value of 18 studies, and the left and right vertical lines represent the 95% CI. Each dashed line perpendicular to the vertical line represents the results of combined studies after excluding corresponding studies; the circle represents the OR value of combined studies, and the left and right boundaries represent the 95% CI.

From the analysis result of the recessive model, there was significant heterogeneity among the 18 included studies. Therefore, we did a subgroup analysis of multiple factors, including ethnicity, source of controls, histological classification, smoking, and gender. The results are shown in **Table 2**. Our subgroup analysis of the different sources of controls mainly included Hospital-based study (HB) and Population-based study (PB). In the Hospital-based study group, the OR was 1.34 (95% CI: 1.04–1.72, I²= 49.9%, P =0.051). However, there was no significant difference in other groups. Metaninf is our method to investigate the influence of each study on the overall meta-analysis summary assessment (36). The results are shown in **Figure 4**. As shown in the results of metaninf, when we omitted each study in turn and merged other studies, the recalculated ORs were not materially altered. Therefore, we believe that our results were statistically steady.

**Table 2:** Results of each subgroup analysis.

Subgroups	N^a^	OR	95% CI	I^2^	P^b^
Source of controls					
HB	8	1.34	(1.04, 1.72)	49.9%	0.051
PB	9	0.91	(0.73, 1.12)	62.4%	0.007
Ethnicity					
European	10	1.12	(0.98, 1.28)	23.9%	0.223
Asian	8	0.97	(0.65, 1.44)	78.9%	<0.001
Gender					
male	4	0.88	(0.51, 1.50)	65.6%	0.033
female	5	1.04	(0.65, 1.66)	50.9%	0.086
Smoking					
Smoke	4	1.09	(0.64, 1.85)	74.8%	0.008
Nosmoke	6	1.00	(0.65, 1.54)	70.0%	0.005
Histological type					
Adenocarcinoma	5	1.01	(0.68, 1.49)	62.3%	0.031
Squamous carcinoma	4	0.79	(0.53, 1.19)	63.4%	0.042
Small-cell carcinoma	2	0.95	(0.57, 1.60)	<0.1%	0.366
Large-cell carcinoma	2	2.27	(0.96, 5.37)	<0.1%	0.590
Total^c^	18	1.08	(0.92, 1.27)	62.2%	<0.001

a The number of studies.

b The p-value for heterogeneity.

c The 18 studies used to analyze the slow versus rapid + intermediate genotypes.

### Publication Bias

**Figure 5:**
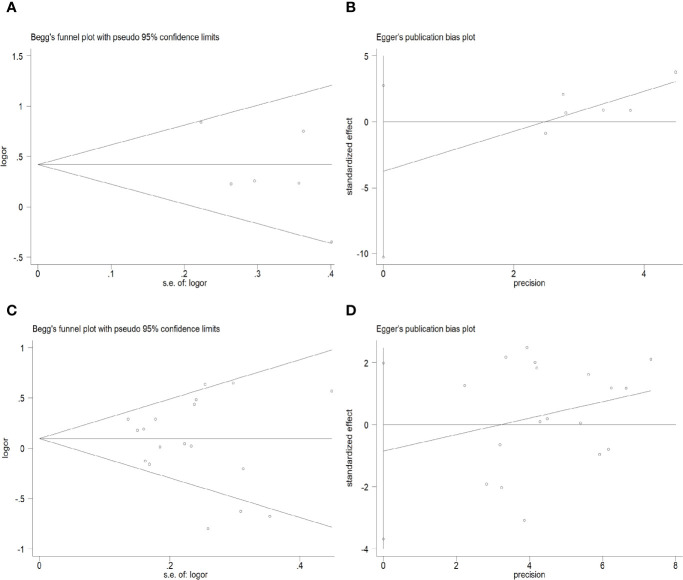
Begg’s funnel plots and Egger’s tests for publication bias in the selection of studies. **(A)** Begg’s Test of the primary model. **(B)** Egger’s test of the primary model. **(C)** Begg’s Test of the recessive model. **(D)** Egger’s test of the recessive model.

As shown in **Figure 5**, there was no obvious publication bias in either the primary model or the recessive model (primary model: Begg’s Test: P=0.707>0.05, Egger’s test: P=0.185>0.05; recessive model: Begg’s Test: P=0.762>0.05, Egger’s test: P=0.533>0.05).

## Discussion

As the most commonly diagnosed cancer in the world, the pathogenesis of lung cancer is unclear. Cigarette smoke and other environmental factors are currently recognized as important causes of lung cancer (37). NAT2 is an important two-phase detoxifying enzyme, which is used for the transformation of exogenous chemicals *in vivo* and is involved in the metabolism of various environmental toxicants (38). Many studies have found the effects of NAT2 polymorphism on the risk of many diseases, including allergic diseases and asthma, and various types of cancer including lung cancer, bladder cancer, and gastrointestinal tumors (7). Polymorphisms of the NAT2 gene locus leads to differences in NAT2 enzyme activity between different individuals, which may be related to the occurrence and development of lung cancer (39). Due to NAT2 gene polymorphism, there are different NAT2 alleles, producing slow, intermediate, or rapid phenotype of NAT2. According to the included studies, we found that the results obtained by different studies were quite different and even contradictory in terms of the impact of NAT2 polymorphism on the risk of lung cancer. Some studies found no effect of NAT2 polymorphism on lung cancer risk (9, 12, 19, 20, 22). In other studies, the slow acetylation phenotype of NAT2 may increase the risk of lung cancer (6, 10, 14, 15, 21, 24, 25). However, other researchers have suggested that rapid acetylation phenotype may increase the risk of lung cancer (16–18, 26). Thus, there is no consensus regarding whether the NAT2 gene is associated with lung cancer. The purpose of this meta-analysis was to investigate the effect of NAT2 polymorphism on lung cancer risk.

In the primary model (slow + intermediate phenotype *vs*. rapid phenotype), the results showed that individuals with non-rapid (slow+intermediate) phenotype had a significantly higher risk of lung cancer. In the comparison of the three NAT2 acetylator statuses, we found that compared with rapid phenotype, both slow NAT2 phenotype and intermediate phenotype significantly increased the risk of lung cancer. Based on the above results, we can infer that the slow allele of NAT2 can reduce the NAT2 enzyme activity. Individuals carrying one or two slow alleles of NAT2 may have a higher risk of lung cancer due to their decreased ability to metabolize various environmental toxicants.

In this review, relevant studies were included in strict accordance with the inclusion and exclusion criteria. Moreover, all the included articles have been evaluated by quality. It can also be seen from the results in **Figure 2** that the results of each included study are not the same, and even different studies get opposite results. Therefore, this study is worthy of meta-analysis. After Prospero was registered, we followed the instructions above strictly and followed the principle of PRISMA and the Cochrane Handbook of Systematic Reviews of Interventions to complete this systematic review. Besides, sensitivity analysis and publication bias analysis were performed. So, we did have a balanced, comprehensive, critical study.

Indeed, similar meta-analyses have been published [Cui D et al. Lung Cancer 2011 (37), Liu C et al. Medicine 2015 (40)]. But compared with these two articles, we still have many new points. First of all, the two studies of Cui D et al. and Liu C et al. only analyzed whether there was a significant difference in lung cancer risk between people with the slow phenotype and those with the other two acetylation statuses. Our research not only analyzed the above model but also analyzed the model of slow+intermediate phenotype *vs*. rapid phenotype and compared the three NAT2 acetylator statuses. The studies of Cui D et al. and Liu C et al. did not get positive results, while our analysis of the models of slow+intermediate phenotype *vs*. rapid phenotype, slow phenotype *vs*. rapid phenotype, and intermediate phenotype *vs*. rapid phenotype all got positive results. Secondly, the article of Liu C et al. (40) focuses on five SNPs in the NAT2 gene, namely C282T, A803G, C481T, G590A, and G857A. In terms of the phenotypes of NAT2, the articles they included were different from ours. Compared with them, we included relatively new studies [Mota et al. J Cancer Res Clin Oncol 2015 (11), Zhang et al. Asian Pac J Cancer Prev 2014 (12)]. In the article by Liu C et al. (40), the stratification factor includes the only source of controls and ethnicity. But our study also included gender, histological type, smoking status, and phenotypes of NAT2. Compared with the article of Cui D et al. (37), we also included different studies [Mota et al. J Cancer Res Clin Oncol 2015 (11), Zhang et al. Asian Pac J Cancer Prev 2014 (12), Sobti et al. DNA Cell Biol 2009 (15), Osawa et al. Asian Pac J Cancer Prev 2007 (17)]. Based on these points, we think our study has something to recommend it. However, there were still some limitations to our study. First, the latest study that meets our requirements is the study of Mota et al. in 2015. There are no more recent studies that can be included. Secondly, although the results were statistically significant, the number of studies included was not convincing enough (only six studies were included). We got positive results, indicating that the relationship between NAT2 and lung cancer is worthy of further study. We believe that the credibility of our study will be improved if there are more studies to analyze the difference of lung cancer risk between the rapid phenotype and the other two acetylation statuses.

## Conclusions

In our analysis, we found that people with NAT2 non-rapid (slow + intermediate) phenotype have a significantly increased risk of lung cancer. We also found that compared with rapid phenotype, both slow NAT2 phenotype and intermediate phenotype significantly increased the risk of lung cancer. Rapid, intermediate, and slow phenotypes represent the speed of acetylation. People with rapid acetylation may have a lower risk of lung cancer. Besides, considering that there were differences in the study population selection and control group classification criteria in various studies, and many studies have suggested that NAT2 polymorphism has effects on lung cancer risk, we believe that NAT2 has a certain influence on lung cancer.

## Data Availability Statement

The original contributions presented in the study are included in the article/**Supplementary Material**. Further inquiries can be directed to the corresponding authors.

## Author Contributions

KZ, AX, and WX analyzed the data and wrote the first draft. RW and SZ designed the study, proofread, and revised the submission. HJ directed the statistical analyses of the data. BZ and PL retrieved documents and extracted data. All authors contributed to the article and approved the submitted version.

## Funding

The fund for the Natural Science Foundation of China (No. 81970051), Excellent Top Talent Cultivation Project of Anhui Higher Education Institutions (gxyqZD2017030), the fund from Reserve candidate for Anhui Province Academic and technical leader, and scientific research fund from Anhui medical university (2020xkj257) supported this research.

## Conflict of Interest

The authors declare that the research was conducted in the absence of any commercial or financial relationships that could be construed as a potential conflict of interest.

The reviewer SF declared a shared affiliation, with no collaboration, with several of the authors, KZ, BZ, HJ, RW, to the handling editor at the time of review.
