# Supplementary material for: Association Between NAT2 Polymorphism and Lung Cancer Risk: A Systematic Review and Meta-Analysis
Source: Front Oncol. 2021 Mar 11;11:567762. doi: 10.3389/fonc.2021.567762 (PMC7991837; doi:10.3389/fonc.2021.567762)
Supplement: Supplementary file 1 [file Table_1.docx]

Table S1 The detailed search strategy

| Database | Search Strategy |
| --- | --- |
| Pubmed | #1 “Lung Neoplasms”[MeSH Terms] OR “Lung Neoplasms” OR “Pulmonary Neoplasms” OR “Neoplasms, Lung” OR “Lung Neoplasm” OR “Neoplasm, Lung” OR “Neoplasms,Pulmonary” OR “Neoplasm,Pulmonary” OR “Pulmonary Neoplasm” OR “Lung Cancer” OR “Cancer, Lung” OR “Cancers, Lung” OR “Lung Cancers” OR “Pulmonary Cancer” OR “Cancer, Pulmonary” OR “Cancers, Pulmonary” OR “pulmonary Cancers” OR “Cancer of the Lung” OR “Cancer of Lung”  #2 “N-acetyltransferase 2” OR “NAT2”  #3 “Polymorphism, Genetic”[Mesh] OR “Polymorphisms, Genetic” OR “Genetic Polymorphisms” OR “Genetic Polymorphism” OR “Polymorphism (Genetics)”  #4 #1 AND #2 AND #3 |
| Embase | #1 'neoplasms, lung' OR 'lung neoplasm'/exp OR 'lung neoplasm' OR 'neoplasm, lung' OR 'neoplasms, pulmonary' OR 'neoplasm, pulmonary' OR 'pulmonary neoplasm'/exp OR 'pulmonary neoplasm' OR 'lung cancer'/exp OR 'lung cancer' OR 'cancer, lung'/exp OR 'cancer, lung' OR 'cancers, lung' OR 'lung cancers' OR 'pulmonary cancer'/exp OR 'pulmonary cancer' OR 'cancer, pulmonary' OR 'cancers, pulmonary' OR 'pulmonary cancers' OR 'cancer of the lung' OR 'cancer of lung'  #2 'n-acetyltransferase 2' OR 'n-acetyltransferase 2'/exp OR 'nat2' OR 'nat2'/exp OR 'n-acetyltransferase 2' OR 'n-acetyltransferase 2'/exp OR 'nat2' OR 'nat2'/exp  #3 'polymorphism, genetic' OR 'genetic polymorphism' OR 'polymorphism' OR 'polymorphism, genetic'/exp OR 'genetic polymorphism'/exp OR 'polymorphism'/exp  #4 #1 AND #2 AND #3 |
| Cochrane Library | #1 (Neoplasms, Lung) OR (Neoplasm, Lung) OR (Neoplasm, Pulmonary) OR (Cancer, Lung) OR (Cancers, Lung) OR (Lung Cancers) OR (Cancer, Pulmonary) OR (Cancers, Pulmonary) OR (Pulmonary Cancers)):ti,ab,kw  #2 (N-acetyltransferase 2):ti,ab,kw OR (NAT2):ti,ab,kw  #3 (Polymorphism, Genetic):ti,ab,kw OR (Polymorphisms, Genetic):ti,ab,kw OR (Genetic Polymorphisms):ti,ab,kw OR (Genetic Polymorphism):ti,ab,kw OR (Polymorphism (Genetics)):ti,ab,kw  #4 #1 AND #2 AND #3 |
