# Supplementary material for: Association Between NAT2 Polymorphism and Lung Cancer Risk: A Systematic Review and Meta-Analysis
Source: Front Oncol. 2021 Mar 11;11:567762. doi: 10.3389/fonc.2021.567762 (PMC7991837; doi:10.3389/fonc.2021.567762)
Supplement: Supplementary file 2 [file Table_2.docx]

**TableS2. Quality evaluation form of the included studies**

| author | selection of study | comparability of groups | ascertainment of exposure and outcomes | NOS score |
| --- | --- | --- | --- | --- |
| Mota | **2** | **2** | **3** | **7** |
| Zhang | **3** | **2** | **3** | **8** |
| Mahasneh | **3** | **1** | **3** | **7** |
| Zupa | **2** | **NA** | **3** | **5** |
| Sobti | **3** | **2** | **2** | **7** |
| Lee | **4** | **2** | **2** | **8** |
| Osawa | **3** | **2** | **3** | **8** |
| Chen | **4** | **2** | **2** | **8** |
| Chiou | **4** | **2** | **2** | **8** |
| Sorensen | **2** | **2** | **2** | **6** |
| Skuladottir | **4** | **1** | **3** | **8** |
| Belogubova | **3** | **NA** | **3** | **6** |
| Wikman | **3** | **NA** | **3** | **6** |
| Hou | **3** | **NA** | **2** | **5** |
| Saarikoski | **4** | **NA** | **2** | **6** |
| Seow | **3** | **1** | **2** | **6** |
| Nyberg | **4** | **NA** | **2** | **6** |
| Martinez | **3** | **NA** | **3** | **6** |
